# Augmentation Rhinoplasty and Centrofacial Lipofilling: Our Experience (ARCL)

**DOI:** 10.3390/jcm13071965

**Published:** 2024-03-28

**Authors:** Mirco Pozzi, Pietro Susini, Davide di Seclì, Michela Schettino, Luca Grimaldi, Roberto Cuomo, Carlos Weck Roxo

**Affiliations:** 1 Unit of Plastic and Reconstructive Surgery, Department of Medicine, Surgery and Neuroscience, University of Siena, 53100 Siena, Italy; susinipietro@gmail.com (P.S.); davidedisecli@hotmail.it (D.d.S.); luca.grimaldi@unisi.it (L.G.); robertocuomo@outlook.com (R.C.); 2 Unit of Plastic and Reconstructive Surgery, CHIREC de Braine L’Alleud Hospital, 1410 Brussels, Belgium; michelaschettino@gmail.com; 3 Instituto Carlos Roxo, Avenida Ayrton Senna n°1850, Rio de Janeiro 22775-003, RJ, Brazil; carloswroxo@hotmail.com

**Keywords:** rhinoplasty, augmentation rhinoplasty, open rhinoplasty, facial rejuvenation, centrofacial lipofilling

## Abstract

**Introduction:** Augmentation rhinoplasty traditionally represents a serious challenge for plastic surgeons. The association with centrofacial lipofilling is a great approach to achieve harmonious, aesthetic results. The aim of this article is to describe our personal association between Augmentation Rhinoplasty and Centrofacial Lipofilling (ARCL) in non-Caucasian patients. **Materials and Methods:** In this study, we retrospectively reviewed patients treated with ARCL at our institution between January 2019 and December 2023. We described our personal approach and technique. At a minimum follow-up time of one year, post-operative pictures were taken, and patients were reassessed, evaluating aspects such as global symmetry, shape and contour of the nose, and facial harmony and rejuvenation; finally, patients’ satisfaction was investigated according to the ROE questionnaire and the modified S-GAIS. **Results:** A total of 307 patients were included in the study. They reported a significant satisfactory aesthetic result in nasal image and facial harmony, as the mean postoperative ROE and S-GAIS score show. None of the grafts extruded or collapsed. Wounds healed without reported major infection. **Conclusions:** This study has demonstrated that ARCL is a safe approach that contributes to improve functional and aesthetic outcomes, has a high patient satisfaction rate, and limited post-operative complications.

## 1. Introduction

Rhinoplasty represents one of the most popular plastic surgery procedures according to the American Society of Plastic Surgeons [1]. This cosmetic procedure aims to restore harmony and symmetry in nasal shape and volume, trying to reach an ideal standard of beauty.

Historically, standards of beauty have been defined on Caucasian people. Thus, the comparison of these ideals to any other patients, also defined as non-Caucasian [2,3,4], often generated unsatisfactory feelings and aesthetic results following plastic surgery procedures.

Specifically, the concept of ethnic nose rhinoplasty rose and started defining a surgical procedure usually performed on non-Caucasian people [5]. It represents a great challenge for plastic surgeons. It aims to reshape the nose according to the patients’ face structure, rather than pursuing an ideal standard of beauty. In particular, in Afro-American patients, the comprehension of facial structure, beauty norms, and psychological background is fundamental while performing rhinoplasty [6,7,8].

Lipofilling is gaining importance among plastic surgeons. It is a safe and effective procedure to obtain filler material, and its use in facial cosmetic procedure is increasing. As stated before, rhinoplasty procedures have to be tailored to the patients’ face, and more often, age-related volume loss and alteration of facial structures (malar hypoplasia, periapical hypoplasia, and microgenia) can affect aesthetic outcome. Due to its central position on the face, rhinoplasty should be associated with treatment of other facial structures to optimize the overall outcome [9,10]. Fat grafting can represent a safe alternative to facial implant or osteotomies, restoring or recreating volumes of the face with long lasting results [11,12,13]. Few studies described the use of lipofilling in combination with rhinoplasty. The aim of this study is to retrospectively analyze and describe our technique, the Augmentation Rhinoplasty and Centrofacial Lipofilling (ARCL), and evaluate the association of these two surgical techniques in achieving harmonic aesthetic results and patients’ satisfaction.

## 2. Materials and Methods

### 2.1. Study Design

We retrospectively reviewed patients who underwent ARCL at our clinic in Rio de Janeiro, Brazil, between January 2019 and December 2022.

The indication for rhinoplasty combined with centrofacial lipofilling was given by the aesthetic analysis of the senior author (CWR), or by specific request of the patient for a change in facial appearance or proportions. Rhinoplasty procedures included primary and secondary rhinoplasties for non-Caucasic patients.

Non-Caucasians were defined as patients presenting with ethnic facial characteristics such as wide and depressed dorsum, ill-defined droopy tip, broad—low projection, alar flaring, and short columella. Moreover, we included non-Caucasian patients who previously presented the aforementioned characteristics but underwent unsatisfactory primary rhinoplasty, seeking for a revision. Indeed, due to the anatomical characteristics, they still required ethic rhinoplasty procedures to treat their previous rhinoplasty.

All patients were aged 18 years or older. All underwent autologous septal or costal cartilage dorsal augmentation rhinoplasty and concurrent centrofacial lipofilling. The minimum follow-up was one year.

**Figure 1:**
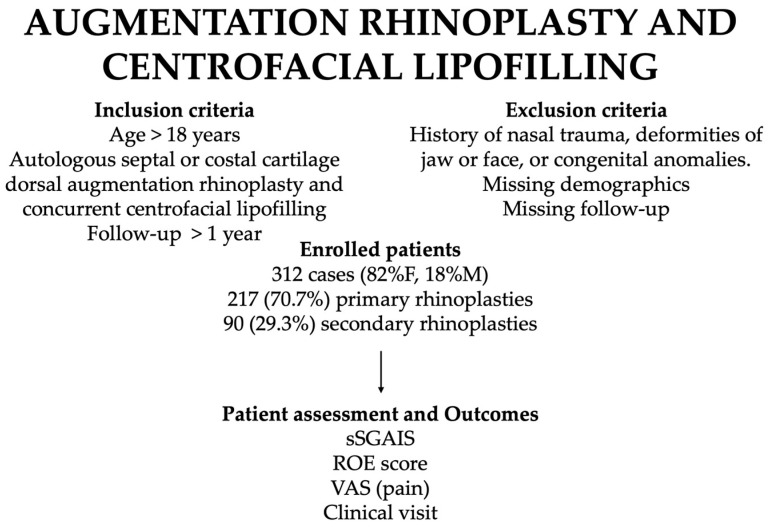
Study diagram to show how patients were assessed.

Patients were excluded in case of history of nasal trauma, deformities of jaw or face, or congenital anomalies. Missing/incomplete data on demographics (age, sex, smoke habit, drug assumption, indication for surgery, and surgical procedure), as well as missing/incomplete follow-up were also considered exclusion criteria. See Figure 1.

Detailed information regarding rhinoplasty and lipofilling procedures and possible complications was shared with all patients at the time of consultation. Informed written consent was obtained from all enrolled patients for analysis and publication of personal data. The article followed the principles of the Declaration of Helsinki.

By reviewing patient records, we analyzed demographics, indications for surgery, surgical procedures, postoperative pain (VAS), and complication rates. The volume of injected fat was quantified for each procedure. Fat grafting specific complications including infection, contour irregularities, cysts formation, and fat emboli were also assessed. Photographic documentation was collected both before the surgeries, during the procedures and at the one-year follow-up. The pictures were taken into standard setting. Specifically, each patient received front, lateral side, and 45 degree shots from a distance of one meter.

No further centrofacial fat grafting procedures were performed during the follow-up period for all patients.

Outcomes of interest consisted of rhinoplasty outcome, such as differences in nasal structures, global symmetry, nose shape and contour, and cartilage donor site morbidity. Furthermore, the results of centrofacial lipofilling were evaluated based on facial harmony, profile vision, femininity, attractiveness, and youthfulness improvement analyzed using the modified 5-points Global Aesthetic Improvement Scale (S-GAIS) . See Table 1.

The ROE consisted of six questions on a scale of 0 to 4, with the highest score related to increased patient satisfaction. The questionnaire was answered anonymously by each patient. The numerical value of each response was added, and the total was divided by 24 and multiplied by 100 to obtain a final result between 0 and 100, where 100 indicated absolute patient satisfaction and 0 indicated absolute disappointment.

The aim of the present paper is to report our experience with primary and secondary rhinoplasty and concurrent centrofacial lipofilling. Therefore, a comparison with alternative rhinoplasty techniques or facial treatments was not performed.

Statistical analysis was performed with SPSS software 2016 (IBM Corp., Armonk, NY, USA) to test for any correlation between patient demographics and ARCL outcome. Statistical significance was defined as *p* < 0.05. The senior author designed and performed the described technique for all patients enrolled in the study.

### 2.2. Operative Technique

The operating strategy involves two surgeries performed in the same operating session. First the rhinoplasty, followed by centrofacial lipofilling. A careful preoperative analysis, including markings, is performed to determine the volume and placement area for the fat graft, with the patient in the standing position, before induction of anesthesia. All patients receive intraoperative antibiotic prophylaxis with amoxicillin and clavulanate. Midfacial fat grafting is always performed at the end of the procedure, after rhinoplasty. The main reason is that augmentation rhinoplasty significantly affects the overall facial harmony. As a result, a more precise facial analysis is only possible later, after rhinoplasty.

### 2.3. Rhinoplasty

The rhinoplasty is performed according to the open rhinoplasty technique by the senior author Carlos Weck Roxo. Technical details have been reported in a previous paper [9]. Briefly, the technique involves a dorsal augmentation open rhinoplasty, with costal cartilage grafts, performed under general anesthesia. The costal cartilage is commonly harvested from the sixth or the seventh rib, with a standard inframammary incision of 1.5 cm. Then, the harvested cartilage is cut and shaped into the required grafts. Moreover, some cartilages pieces are cut with a knife to create diced cartilages for dorsum augmentation. An inverted “V” incision of the columella allows for lower lateral cartilages exposure. Then, we proceed with SMASS debulking. Specifically, we separate the SMASS layer (below) and the Subdermic layer (on top), in an avascular plane, preserving vessels. The dissection continues over the upper lateral cartilages and bony dorsum up to the root of the nose.

After adequate dorsal exposure, the need for dorsal augmentation is assessed. Since most ethnic patients present with a low and wide nasal dorsum, with small nasal bones, low-high lateral osteotomies are usually performed, followed by subperiosteal diced cartilage dorsal augmentation until specifically needed. Then, the septoplasty is performed, and extended spreader grafts (ESG) and septal extension graft (SEG) are settled to stabilize and correct the septum and the tip. The lower lateral cartilages are reshaped, while inter-domal or trans-domal sutures allow for fine tip definition. Articulated alar rim grafts (AARGs) are settled to resolve external nasal valve collapse and help prevent nasal contracture and retraction. Finally, if necessary, onlay grafts can be performed for further adjustment of projection and tip definition. PDS 5.0 suture secures our grafts in place. Then, basal view inspection of the nose is performed to evaluate any alar base abnormalities. Eventually, alar base resection is realized to fix the excess flaring of the alar rims. External thermoplastic nasal splint is set and kept in place for one week.

### 2.4. Centrofacial Lipofilling

At the end of the rhinoplasty, we proceed with centrofacial lipofilling. The modified Klein solution (1:1,000,000) is infiltrated at the donor site, which is typically represented by the inner thigh using a 2.4 mm diameter cannula. At least 40 cc of fat are harvested and then decantated for 20 min to separate adipose tissue from oil and fluids. In addition, some fat is treated as Microfat for the finest definition. Microfat graft preparation is performed by transferring the harvested fat through the Microfat transfer 20 times. Then, both fat and Microfat is passed to 1 mL syringes for subsequent injection.

**Figure 2:**
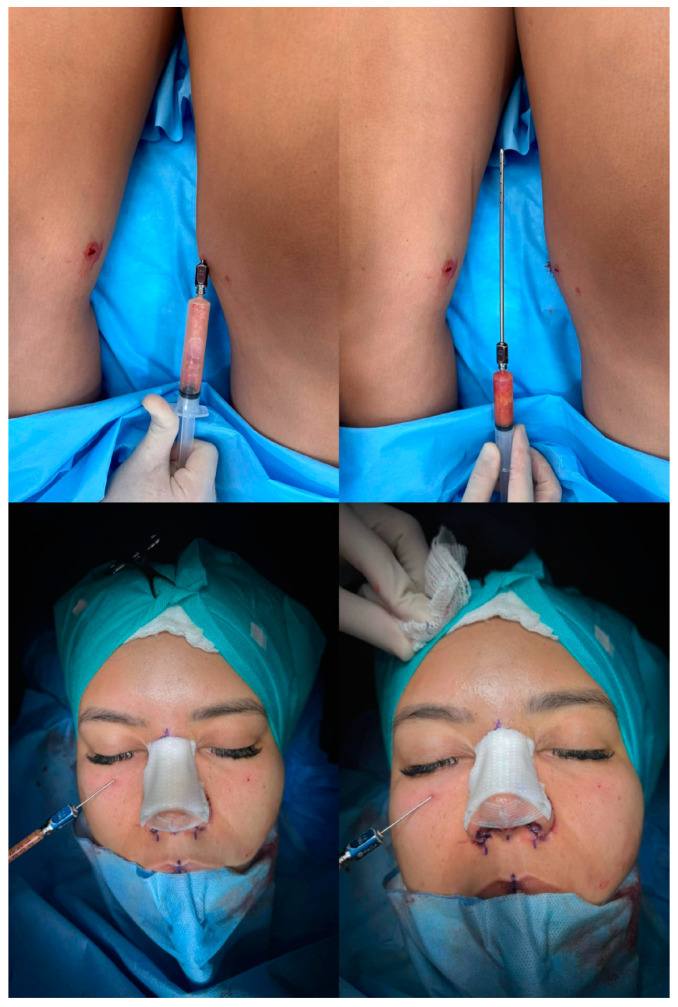
ARCL procedure. Liposuction at the level of the inner thigh and lipofilling at the end of rhinoplasty are shown.

Fat grafting addresses the tear through deformity, malar regions, nasolabial folds, and the chin. The surgeon’s experience determines the volume of fat grafting in each area. To improve chin projection, a slight overcorrection is suggested in consideration of the higher absorption rate (almost 40%) that typically affects this area. The average volume that is necessary for chin augmentation is variable. A minimum chin augmentation requires 6 cc, up to 10–12 cc for greater ones. By contrast, overcorrection is not necessary at the tear through deformity. For tear through deformity correction, 1 cc of fat graft is typically injected at the deep layer, above the periosteum, plus another layer, intramuscular, of 1 cc of Microfat. Microfat may also be used to treat hollow eye deformity. Similarly, nasolabial fold correction and malar region augmentation is normally accomplished by injecting 2 cc of fat per area in each side. See Figure 2.

Amoxicillin and clavulanic antibiotic are prescribed for seven days. Oral Isotretinoin is started one week after surgery and continued for at least three months to prevent nasal inflammation and edema. Follow-up is regularly performed up to at least one year after surgery.

## 3. Results

### 3.1. Study Population

From January 2019 to December 2023, 307 patients underwent ARCL at our clinic in Rio de Janeiro. The senior author performed all surgeries. The average age was 43.2 years old (range 22–68); 82% were females, 18% were males. Twenty-one (6.8%) were smokers, and oral steroids were taken by twelve (3.9%).

A primary rhinoplasty was realized in 217 (70.7%), while secondary rhinoplasty was realized in 90 (29.3%). Fat grafting was directed at the tear trough deformity 250 (81.44%), with an average fat volume of 1.1 cc (range 0.5–1.5 cc) on each side, malar region in 269 (87.6%), with an average fat volume 2.2 cc (range 1.75–2.5 cc) on each side, nasolabial folds in 190 (61.9%), average fat volume 4.2 cc (range 3–5 cc) on each side, and the chin in 230 (75%) average fat volume 8.5 cc (range 5–12 cc). The minimum follow-up was 12 months for all patients (average follow-up 14, range 12–24 months).

### 3.2. Outcomes

Successful ARCL has been realized in all enrolled patients. No significative difference in complications and outcomes was noticed among different patient populations, suggesting that this surgical strategy is safe and effective in various clinical settings. Specifically, no statistically significative difference (*p* value > 0.05) was observed testing the relationship between complication rates and patients’ demographics, such as smoking status (smoker vs. non-smoker), reason for surgery (primary vs. secondary rhinoplasty), and surgical procedure (tear through deformity vs. nasolabial folds vs. malar region lipofilling).

Regarding the ethnic rhinoplasty specific complications, we did not observe any case of nasal contracture, cartilage grafts extrusion or collapse, and skin infection or necrosis. Minor infections were developed by four patients, successfully treated with oral antibiotics. For cartilage donor site morbidity, no wound dehiscence, infection, or pneumothorax occurred. Secondary surgeries were not required in any cases.

Regarding centrofacial lipofilling, no infection, major contour irregularities, cysts formation, and fat emboli occurred, only one case of fat show in lower eyelid. Five patients complained of facial long edema, but all healed in three months without requiring specific treatments.

Post-operative pain according to VAS score was recorded on post-operative day one and day seven. The value was 4.1 (range 1–5) and 1.9 (range 1–3), respectively.

At the one year follow-up, ARCL outcomes were evaluated by the surgeon and patient’s satisfaction was investigated according to the modified 5 points S-GAIS scale and the ROE questionnaire.

According to the modified S-GAIS scale, the average score for facial harmony was 1.3 (range 1–3), profile vision 1.2 (range 1–2), femininity 2.4 (range 1–4), attractiveness 2.2 (range 1–4), and youthfulness improvement 1.8 (range 1–2).

For the ROE questionary, satisfactory aesthetic results were reported by all patients, revealing a mean postoperative ROE score of 93.2.

Noses projection appears improved, including a defined tip. The nasal augmentation is preserved at the dorsum. No facial asymmetries are observed between the various structures. Fat grafting results are satisfactory, showing overall facial harmony improvement.

**Figure 3:**
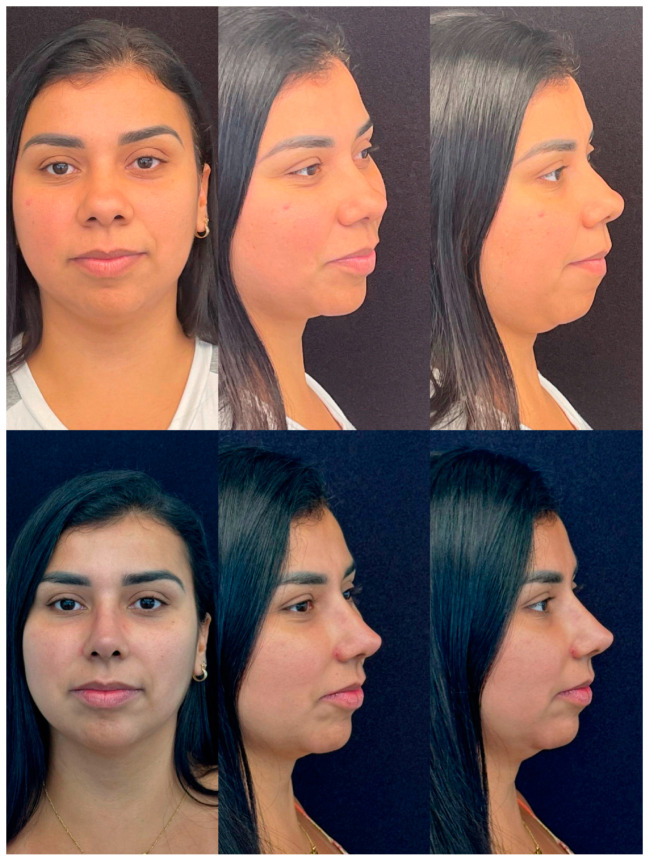
18 months follow-up; 28 old year female. Primary rhinoplasty and centrofacial lipofilling.

**Figure 4:**
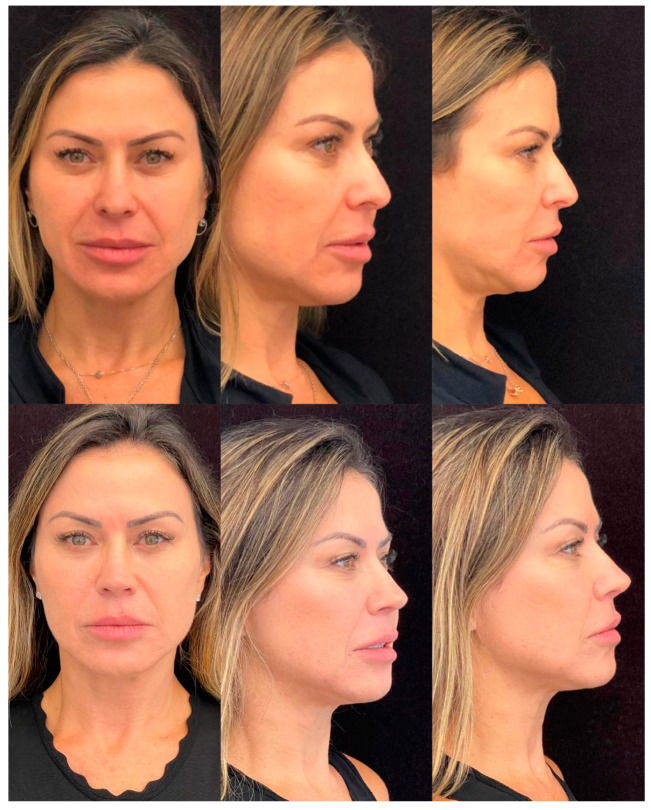
12 months follow-up, 39 old year female. Secondary rhinoplasty including extended spreader grafts (ESG) and septal extension grafts (SEG) and centrofacial lipofilling.

Some results are shown in Figure 3 and Figure 4.

## 4. Discussion

The art of rhinoplasty is constantly evolving. Although new surgical approaches, such as preservation rhinoplasty, have been developed in recent times, we believe that the open technique is the most appropriate in ethnic noses [14,15,16,17]. In fact, an open approach offers the best visualization, allowing accurate placement of the grafts. An adequate aesthetic reconstruction could be more difficult and limited with classic intranasal techniques [15,16].

Specifically, in augmentation rhinoplasty the use of a strong structural support by various extended spreader grafts (ESP) and septal extension graft (SEG) resulted in significantly better long-term results with maintenance of projection and reduced tip deformities. For this reason, we recommend reconstruction with this kind of graft. In addition, placement of a second partial SEG to delineate the tip makes the structure more stable and straight [8].

Ethnic rhinoplasties focus on the specific nasal characteristics of certain ethnic groups and the adherence to their aesthetic standards. In the context of ethnic rhinoplasty, the Asian population cannot be overlooked [17,18,19]. In the case of Asian rhinoplasties, Wan R. et al. [20] addresses the unique challenges associated with the limited availability of supplemental autologous cartilage, a factor that can significantly impact surgical outcomes. It proposes the use of fresh frozen cadaveric cartilage as an effective and safe alternative, specifically tailored to meet the needs of Asian patients.

Furthermore, it is crucial to achieve facial harmony that does not distort the physiognomy of non-Caucasian patients [21,22,23,24].

Centrofacial lipofilling holds pivotal significance in ethnic facial aesthetic surgery, precisely sculpting and enhancing features for diverse patients. This technique, blending artistry with medical precision, addresses age-related volume loss, ensuring natural outcomes. Particularly crucial in non-Caucasian populations, it harmonizes facial structures, emphasizing individualized beauty norms. By tailoring augmentation to patients’ unique facial characteristics, centrofacial lipofilling not only achieves aesthetically pleasing results, but also contributes to patients’ cultural identity and satisfaction [25]. In the field of facial cosmetic procedures, this approach is a key tool for achieving personalized and culturally sensitive results for different patients [26,27,28,29].

Moreover, according to our experience, lipofilling associated with rhinoplasty promotes anti-inflammatory effects, aiding in facial recovery and optimizing the overall outcome for enhanced patient well-being [10,11,12,13].

Our study retrospectively analyzed patients who underwent open Augmentation Rhinoplasty and Centrofacial Lipofilling (ARCL) in Rio de Janeiro between January 2019 and December 2022. The research focused on tailoring rhinoplasty to non-Caucasian patients, emphasizing ethnic considerations. Lipofilling, an increasingly utilized technique, was employed to address age-related volume loss and enhance overall aesthetic outcomes. The methodology involved a two-stage procedure, starting with rhinoplasty and concluding with centrofacial lipofilling.

The comprehensive evaluation encompassed aesthetic improvements, nasal projection, and facial harmony preservation. Noteworthy, fat grafting demonstrated satisfactory results, contributing to overall facial enhancement in the long term follow up as reported by the positive scores in modified S-GAIS and ROE questionnaires, indicating high patient satisfaction. Lastly, no significant complications were observed in ethnic rhinoplasty and lipofilling, with only minor infections promptly treated.

Study limitations include the absence of a direct comparison with alternative treatment options including rhinoplasty techniques and facial procedures such as face lift, sniff, blepharoplasty, and aesthetic medicine treatments. Moreover, we did not compare patients who underwent rhinoplasty alone vs. rhinoplasty + lipofilling. However, in our experience, each treatment may represent a viable choice depending on specific facial anatomy, patients need and request, including psychological considerations. Therefore, a true comparison may have inherent biases. Furthermore, it is difficult to achieve scientifically.

## 5. Conclusions

We propose our approach (ARCL), employed on a large number of ethnic noses, to achieve long-term results and high patients’ satisfaction. This study has demonstrated that the association between augmentation rhinoplasty and centrofacial lipofilling is a safe procedure that contributes to improve functional and aesthetic outcomes. In conclusion, the ARCL technique, combining ethnic rhinoplasty and lipofilling, was proved to be effective in achieving harmonious aesthetic outcomes for non-Caucasian patients. The study’s thorough methodology, including long-term follow-ups and objective assessments, strengthens its contributions to the field of plastic surgery. Future research may further explore variations in patient demographics and refine techniques for even more personalized and successful outcomes.

## Figures and Tables

**Table 1:** The modified 5-points Global Aesthetic Improvement Scale (S-GAIS). Each parameter presented a preoperative baseline score of 2 points, then it was evaluated based on the following scale: 1 = very much improved, 2 = much improved, 3 = improved, 4 = no change, 5 = worse.

modified 5-points Global Aesthetic Improvement Scale (S-GAIS)	
1 = very much improved, 2 = much improved, 3 = improved, 4 = no change, 5 = worse	
Facial harmony	1.3 (range 1–3)
Profile vision	1.2 (range 1–2)
Femininity	2.4 (range 1–4)
Attractiveness	2.2 (range 1–4)
Youthfulness	1.8 (range 1–2)

## Data Availability

Data are available in the database of the instituto Carlos Roxo, Avenida Ayrton Senna n°1850, Rio de Janeiro RJ 22775-003, Brazil.
